# Impact of neuromodulation on excessive daytime sleepiness: a narrative review

**DOI:** 10.3389/fpsyt.2025.1545206

**Published:** 2025-10-16

**Authors:** Mengmeng Li, Lingfang Feng, Wen Pan, Xiaobin Zhang, Xiangdong Du, Zhe Li

**Affiliations:** Suzhou Guangji Hospital, The Affiliated Guangji Hospital of Soochow University, Suzhou, China

**Keywords:** excessive daytime sleepiness, noninvasive neuromodulation, invasive neuromodulation, cortical arousal, top-down pathway

## Abstract

Excessive daytime sleepiness (EDS), a global health issue, impairs daily functioning and increases the risk of accidents. Neuromodulation, which can adjust cortical excitability, has emerged as a promising EDS treatment. Although only a few studies have been conducted on this topic and sample sizes were consistently small, the available literature shows that anodal transcranial direct current stimulation or high frequency transcranial alternating current stimulation has been shown to reduce EDS caused by a variety of disorders. Moreover, high frequency repetitive transcranial magnetic stimulation (rTMS) over the left dorsolateral prefrontal cortex or low frequency rTMS targeting the right dorsolateral prefrontal cortex or posterior parietal cortex has demonstrated significant positive effects in the treatment of daytime sleepiness. Moreover, non-invasive neuromodulation has been found to provoke a net gain of cortical arousal, which is linked to the modulation of cortical activity by reducing slow-frequency (δ and θ) activity while enhancing faster frequencies (β1 and β2). Invasive neuromodulation, such as deep brain stimulation, has positive effects on sleep regulation in Parkinson’s disease patients, which may contribute to an improvement in EDS. Similarly, vagus nerve stimulation has demonstrated potential benefits for patients with epilepsy, especially those experiencing EDS or co-morbid narcolepsy. Noninvasive neuromodulation for the treatment of EDS presents a promising horizon of opportunities to enhance current therapeutic modalities. However, further research is warranted to refine treatment protocols and validate outcomes through objective measures.

## 1 Introduction

Awake and sleep states represent fundamental physiologic processes to maintain a healthy life and social function. Excessive daytime sleepiness (EDS) is characterized by a persistent struggle to maintain wakefulness and alertness during daytime and is mainly manifested as overwhelming drowsiness, unintended lapses into sleep, sudden sleep episodes, recurrent naps, excessive sleep needs, and prolonged sluggishness upon waking, which may be accompanied by automatic behavior, confusion, fatigue, lack of energy, and impaired cognitive function (1). Epidemiologic studies throughout the world, have shown that >20% of the population report excessive sleepiness during the daytime (2). As a major global public health concern, EDS affects a wide range of individual performances, resulting in reduced learning ability and poor performance in family and professional roles (3). Furthermore, EDS is a contributing factor to the annual incidence of motor vehicle accidents and associated deaths (4). The main causes of EDS are numerous and include insufficient sleep, central hypersomnolence (narcolepsy, idiopathic hypersomnia, and Kleine-Levin syndrome), other sleep disorders (insomnia, obstructive sleep apnea [OSA], and circadian rhythm sleep-wake disorders), a medical or neurologic disorder, medication or substance abuse, and psychiatric disorders (1, 5).

Owing to the complexity of the etiology for EDS there are multiple phenotypes, which depend on the co-morbidity, that make treatment challenging. Actively treating the primary disorder is the first task for EDS patients with a clear cause. A comprehensive approach that combines pharmacologic and non-pharmacologic treatments may lead to better therapeutic outcomes. Medications for EDS include amphetamines and other types of stimulants (modafinil and pitolisant), with limited efficacy and adverse side effects (5). Non-pharmacologic management, especially lifestyle intervention, adjusting bedtime and awakening time, and light therapy, are the basic measures which are also limited by adherence issues (6, 7). So, there is an urgent need for complementary safe strategies in the treatment of EDS.

Neuromodulation, which adjusts cortical excitability, is an emerging treatment approach that has gradually attracted attention in the field of sleep disorders (8). EDS is closely related to sleep and arousal neural networks, encompassing the ascending reticular activation system (ARAS) in the brainstem, which sends projections to the thalamus and cortex, as well as the top-down cortico-thalamic pathway that modulates brain activity and vigilance (9). In this context neuromodulation may represent an interesting treatment that helps improve EDS by inhibiting, stimulating, modifying, or regulating neural networks. Indeed, studies exist that have explored the impact of neuromodulation on EDS. For example, published research reviews have demonstrated that noninvasive brain stimulation (NIBS) improves sleep quality and daytime sleepiness in insomnia patients (10, 11).

**Figure 1:**
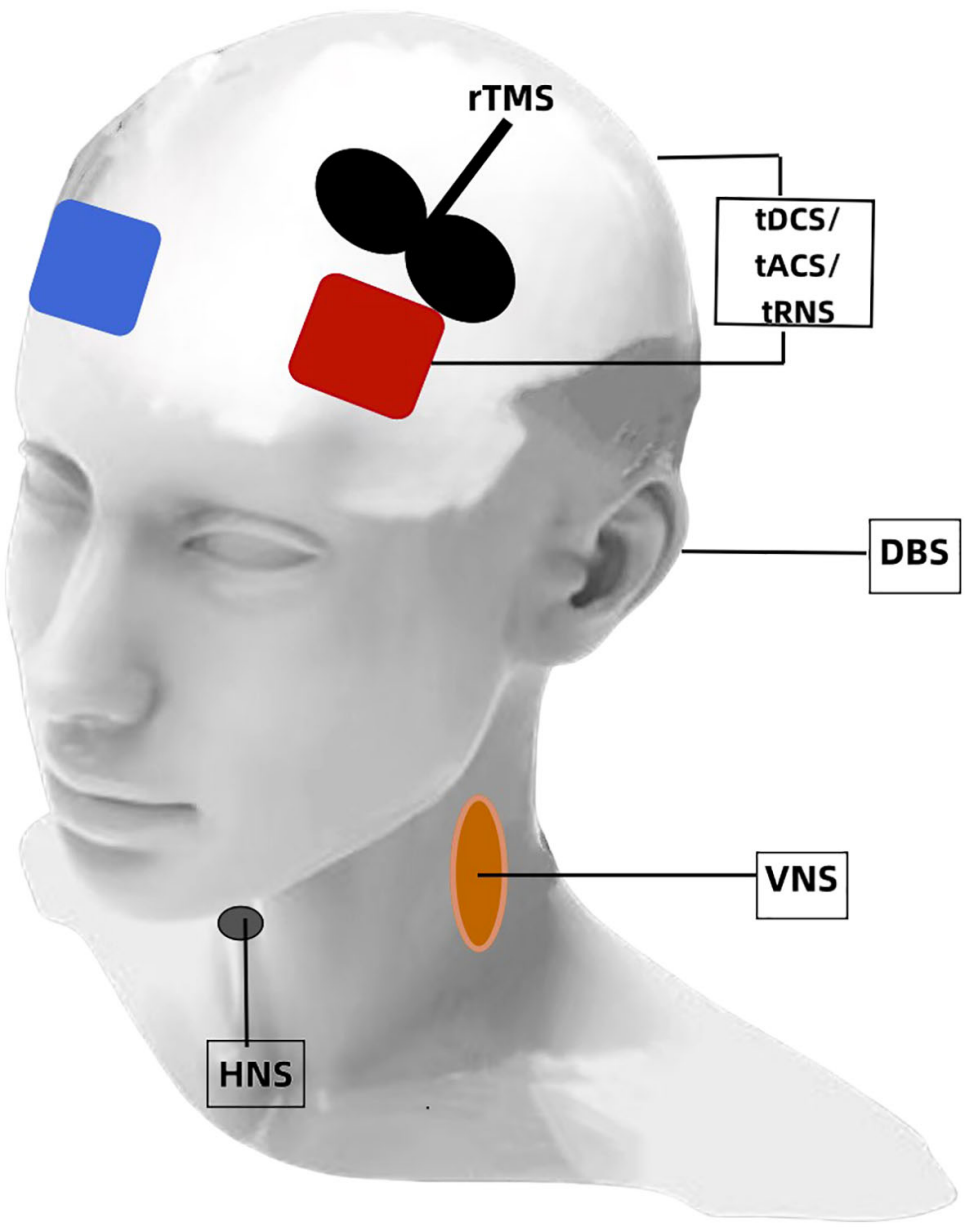
A depiction of neuromodulation techniques for EDS. tDCS, transcranial direct current stimulation; tACS, transcranial alternating current stimulation; tRNS, transcranial random noise stimulation; rTMS, repetitive transcranial magnetic stimulation; DBS, deep brain stimulation; VNS, vagal nerve stimulation; HNS, hypoglossal nerve stimulation.

More specifically, neuromodulation techniques are noninvasive and invasive (**Figure 1**). NIBS includes transcranial electrical stimulation (tES), repetitive transcranial magnetic stimulation (rTMS) (12, 13), and electroconvulsive therapy (ECT). A low intensity electrical current is delivered to the scalp during tES that results in localized changes in cortical excitability and the likelihood of action potential generation (14). The two predominant forms of tES are transcranial direct current stimulation (tDCS) and transcranial alternating current stimulation (tACS). Neuronal resting membrane potential and cortical activity is modulated during tDCS by a constant weak current (0.1–2 mA stimulation) from one electrode to another (15, 16). Anodal stimulation exhibits excitatory effects, while cathodal stimulation exhibits inhibitory effects. Endogenous brain oscillations are modulated during tACS and cortical excitability is altered by applying a sine wave and biphasic alternating current to cortical neurons (17). Transcranial random noise stimulation (tRNS), a novel electrical stimulation method that generates a random noise electrical oscillation spectrum, affects electrical brain activity (18). Electrostatic therapy, as another novel form of tES, creates electrostatic fields through the use of skin-applied patches (19). A swiftly fluctuating magnetic field is utilized during rTMS that penetrates the skull, thereby generating brief electric current surges in the brain to alter cortical functioning (11, 20). rTMS differs from tES because it not only modulates cortical excitability but also directly induces neuronal action potentials (11, 20). ECT is a procedure during which electrical impulses are applied to the head to trigger a seizure (21). Invasive neuromodulation mainly encompasses techniques, such as deep brain stimulation (DBS) and vagal nerve stimulation (VNS). The DBS technique involves implanting electrodes surgically in specific brain regions for delivering controlled electrical impulses and modulating neuronal activity (22). VNS involves subcutaneous surgical implantation of a small pulse generator in the left thoracic region that is programmed for long-lasting intermittent electrical stimulation to the vagus nerve. Transcutaneous auricular VNS (taVNS) allows for noninvasive stimulation of the vagus nerve by a stimulator attached to the auricular conch (23). Hypoglossal nerve stimulation (HNS) is recognized as an alternative to continuous positive airway pressure (CPAP) for patients with OSA. This procedure involves the surgical placement of an electrical stimulator connected to a sensor on the inspiratory intercostal muscles and a lead on the hypoglossal nerve (24).

Although neuromodulation techniques have shown potential in the field of sleep disorders, there have been no relevant reviews published on the neuromodulation treatment for EDS. Herein the application and effectiveness of NIBS (tES and rTMS) and invasive brain stimulation (DBS and VNS) for EDS are reviewed. This work provides a comprehensive perspective of existing research and helps guide future studies and offers some therapeutic perspectives.

## 2 Methods

A literature search was performed in PubMed and Web of Science of studies involving neuromodulation treatment of EDS published prior to August 1, 2024. A blend of the following search terms was used: neuromodulation; brain stimulation; noninvasive brain stimulation; invasive neuromodulation; excessive daytime sleepiness; EDS; hypersomnia; drowsiness; and somnolence. The initial search resulted in 155 studies in PubMed and 241 studies in Web of Science. Studies were included if they: (1) involved human subjects; (2) applied neuromodulation techniques; (3) reported outcomes related to EDS; (4) were published in English. Excluded were: animal studies, reviews, editorials, and studies not reporting EDS-specific outcomes. Fifty-five articles were included in this review. The studies consisted of case reports, randomized controlled trials, and retrospective and prospective studies. There were 16 studies on tDCS, seven studies on rTMS, two studies on tACS, one study on tRNS, one study on electrostatic therapy, and two studies on transcutaneous auricular (ta)VNS. There were nine studies on DBS, 5 studies on VNS, and 13 studies on HNS. This narrative review aimed to provide a comprehensive overview rather than a systematic meta-analysis. Nevertheless, we adhered to a structured search and selection process to minimize bias. The selection process involved screening titles and abstracts, followed by a full-text review of potentially eligible articles by two independent authors. Discrepancies were resolved through discussion.

## 3 Results

### 3.1 Effects of neuromodulation on EDS due to sleep deprivation in healthy subjects

Sleep deprivation can increase daytime sleepiness. A multidimensional study confirmed the ability of bifrontal anodal tDCS to reduce sleepiness in sleep-deprived subjects (25). In the single day experiment, 23 participants underwent a within-subject sham-controlled stimulation protocol. Levels of sleepiness were evaluated before and after active/sham stimulation using physiologic (electroencephalogram [EEG] recordings during Maintenance of Wakefulness Test [MWT]), behavioral (psychomotor vigilance task [PVT]), and subjective (Karolinska Sleepiness Scale [KSS] and Visual Analogic Scale for Global Vigor [VAS-gv]) measures. The participants noticed that active stimulation reduced physiologic sleepiness, preventing vigilance drop, and consistently perceived decreased sleepiness on self-report scales, although subjective measures lacked statistical significance. In a previous pilot study, seven subjects underwent active tDCS, while eight received sham tDCS when cognition declined during at least 30 h of sleep deprivation. All participants were required to complete the PVT, Stanford Sleepiness Scale (SSS), and KSS every 2 h on the day of sleep deprivation. Compared to sham treatment, active tDCS improved subjective drowsiness following acute sleep deprivation (26). Similarly, in a comparison study involving the effects of tDCS and caffeine on vigilance during 30 h of extended wakefulness, tDCS resulted in better subjective ratings of sleepiness (27).

It is worth mentioning that two studies conducted by Frase et al. (28) involving healthy humans aimed to demonstrate that tDCS-induced cortical excitability changes before sleep could affect total sleep time (TST). The key finding was that bi-frontal cortical anodal tDCS decreases TST without disturbing overnight memory consolidation (29). This finding added to the evaluation of tDCS as a safe technique. In addition, applying brain stimulation during sleep entrains specific frequencies to alter sleep architecture (30). However, a study that investigated cathodal tDCS during rapid eye-movement (REM) sleep in healthy individuals resulted in no significant changes in sleep parameters and sleepiness scales (31).

In summary (**Table 1**), three studies investigated the impact of neuromodulation (all tDCS) on EDS caused by acute sleep deprivation in healthy individuals, all three of which concluded that tDCS improves daytime sleepiness. The stimulations were performed during daytime and subjective questionnaire scores were mainly adopted. One study utilized bilateral anodal tDCS (F3, F4) stimulation and the other two studies used unilateral anodal tDCS (F3) stimulation. The difference between the two stimulation methods was that one stimulation method was performed when cognitive function declined after acute deprivation and the other stimulation method was applied immediately after acute deprivation. Furthermore, two studies involving healthy individuals demonstrated that bilateral anodal stimulation at FP1 and FP2 before sleep reduced the TST. A study involving cathodal tDCS stimulation at F4 during the REM period at night in healthy individuals had no effect on sleepiness. Therefore, anodal tDCS stimulation of the prefrontal cortex (FP1/FP2/F3/F4, 1–2 mA) is a promising treatment approach for EDS resulting from acute sleep deprivation in healthy individuals.

**Table 1:** Effects of neuromodulation on EDS due to sleep deprivation in healthy subjects.

Study	Study design	Method	Target area	Protocol	Assessment	Results
Healthy individuals with sleep deprivation						
Valentina et al., 2023 (25)	RCT	tDCS	Two anodes over F3 and F4, 2 cathode over temporooccipital	1.5 mA, 2×15 min, at daytime	EEG, PVT, KSS, VAS-sleepiness	Reduced physiological sleepiness and perceived sleepiness
Cheng et al., 2021 (26)	RCT	tDCS	Anode on the F3, cathode on the F4	2 mA, 30 min, when cognition declined	SSS、KSS	Improved in subjective drowsiness
McIntire et al., 2014 (27)	RCT	tDCS	Anode on the F3, cathode on the contralateral bicep	2 mA, 30 min, after 30 h of extended wakefulness	Side-effects questionnaire	Decreased in drowsiness
Healthy individuals						
Frase et al., 2016, 2022 (28, 29)	RCT	tDCS	Anode on the FP1 and FP2, cathode on the P3 and P4	2 mA, 2 × 13 min, prior sleep	PSG	Decreased in TST
Johnson et al., 2018 (31)	RCT	tDCS	Cathodal electrode over F4 and the reference anodal over the contralateral upper forearm	0.4 mA, 5 Hz/0.75 Hz, 25 min, during REM sleep	SSS	No significant changes in SSS

### 3.2 Effects of neuromodulation on EDS in patients with sleep disorders (insomnia, OSA, narcolepsy and idiopathic hypersomnia)

#### 3.2.1 Insomnia patients

Daytime sleepiness is the most common daytime symptom in insomnia patients. Four studies tested the effect of tES on EDS in insomnia patients. Two of the studies focused on tDCS. Frase et al. (28) reported that bifrontal tDCS decreased the TST in healthy individuals. In a corollary study, the impact of the same tDCS protocol on insomniacs was examined, however, a decrease in the TST did not occur (32). It was proposed that persistent hyperarousal processes in patients with insomnia is a potential mechanism preventing the arousal-inducing effect of anodal tDCS observed in controls. However, in another study among student athletes with sleep disturbances, 2 mA frontal cathodal tDCS at FPz and Pz improved subjective sleep (33). The latest prospective study (34) investigated the impact of tACS in nine patients with insomnia. The stimulation was applied simultaneously and bilaterally at F3/M1 and F4/M2 electrodes before sleep. There were significant improvements in sleep quality and daytime sleepiness (34). In a single arm open-label study, the efficacy of electrostatic therapy on daytime symptoms was evaluated in insomnia patients (35). Thirty subjects underwent a 6-week electrostatic therapy regimen using two 20 mm × 30 mm patches throughout sleep (35). Paired comparisons showed significant Epworth Sleepiness Scale (ESS) score reductions after 3 and 6 weeks. Moreover, after 6 weeks the average ESS score decreased from 7.93 to 5.83, suggesting a transition from mild-to-no daytime sleepiness. Only one study evaluated the influence of rTMS on EDS due to insomnia. In the TMS-EEG study, after 14 days of 1 Hz rTMS targeting the right posterior parietal cortex, the ESS score dropped significantly post-treatment and 1 month later (36). Additionally, there was one study concerning the effect of taVNS on EDS in insomnia patients. The stimulating region was the auricular concha, which is innervated by the afferent auricular branch of the vagus nerve (ABVN). Thirty-six patients received two sessions of 20 Hz dilatational wave stimulation twice a day and the questionnaire evaluation of EDS was shown to have significantly improved (37).

#### 3.2.2 Patients with OSA

Application of electrical stimulation to the dilators of the upper airway is a novel treatment for OSA (24). Many retrospective and/or prospective studies have reported the effects of HNS on the ESS score in OSA; all reports showed that EDS was reduced (38–49). Two selected cases reported that combined hypoglossal and phrenic nerve stimulation (PNS) improve sleep apnea and daytime sleepiness (50). Transcutaneous electrical stimulation (TES) is under investigation as a noninvasive option for the upper airway but there are currently no reports on the impact of TES on EDS in OSA patients.

#### 3.2.3 Narcolepsy and idiopathic hypersomnia patients

Narcolepsy is a central disorder of hypersomnolence. The clinical presentation of narcolepsy includes EDS, cataplexy, sleep paralysis, and hypnagogic or hypnopompic hallucinations (5). Idiopathic hypersomnia (IH) also results in EDS in the absence of another identified cause (5). Two studies have reported the efficacy of tES in patients with narcolepsy and IH. A recent pilot study involved 29 subjects in whom tDCS/tRNS was administered over 3 days, resulting in a modest decrease in ESS scores, but could not be distinguished from the placebo response (51). The study did not establish any significant clinical benefits of single tDCS or tRNS sessions on EDS in hypersomnia patients. In contrast, the effects of 4 weeks of tDCS on IH showed that EDS was significantly reduced; all patients were assigned to receive anodal tDCS treatment (52). The evaluation of subjective daytime sleepiness and attentional domain were assessed by the Attentional Network Task (ANT) at baseline and after treatment. In addition to amelioration of subjective EDS, there was an objective improvement in the attentional domain in all conditions of the ANT, indicating that tDCS may foster the management of EDS in IH and also improve the attentional domain.

Only one case report described the efficacy of narcolepsy-like symptoms with high-frequency rTMS (53). A 14-year-old girl who had EDS, cataplexy, and hypnagogic hallucinations for 5 years, received 10 Hz rTMS over the left dorsolateral prefrontal cortex (DLPFC). The ESS score decreased from 15 to 7 post-stimulation, reflecting a significant improvement in EDS. Furthermore, she EDS and hypnagogic hallucinations, but not cataplexy, had recurred at the follow-up evaluation the following year (53).

Interestingly, VNS has been reported in the treatment of narcolepsy. The study included patients with narcolepsy who underwent VNS because of depression or epilepsy and were compared to controls without narcolepsy who also received VNS for depression or epilepsy (54). Compared to baseline, patients with narcolepsy had a notable improvement in ESS after 3 and 6 months and a tendency towards a reduction in cataplexies (54). However, no significant enhancement in ESS was detected in patients without narcolepsy.

In conclusion, many kinds of sleep disorders can lead to EDS (**Table 2**). Some studies have reported that tES targeting F3/F4/FP1/FP2 and low frequency rTMS at F4 improves EDS associated with insomnia (10); the improvement in ratings from low-frequency rTMS lasted 1 month (36). There are few studies involving the effect of these treatments on EDS in insomnia patients and the research methods and results are diverse and inconsistent, so further studies are needed. For EDS related to OSA, HNS stimulation improves sleepiness while improving OSA. Anode F3 stimulation with tDCS and high frequency rTMS may be a possible option for central hypersomnolence. Fortunately, the effects of high frequency rTMS can last up to 1 year (53). Moreover, VNS is a possible treatment option for narcolepsy.

**Table 2:** Effects of neuromodulation on EDS in patients with sleep disorders.

Study	Study design	Method	Target area	Protocol	Assessment	Results
Insomnia patients						
Frase et al., 2019 (32)	RCT	tDCS	Anode on the FP1 and FP2, cathode on the P3 and P4	2 mA, 2 × 13 min, prior sleep	PSG	No effect in TST
Charest et al., 2019 (33)	RCT	tDCS	Cathodal electrode at FPz and a return electrode at Pz	2 mA, 20 min, 2 day, at daytime	PVT, ESS	Decreased in ESS
Motamedi et al., 2023 (34)	RCT	tACS	Anodal electrodes at F3 and F4, cathodal electrode in the mastoids, bilaterally	0.75 mA, 0.75 Hz, 5 min, prior sleep	ESS	Decreased in ESS
Dai et al., 2023 (35)	RCT	Electrostatic therapy	Temple	1,500 ± 3% V, during nighttime sleep	ESS	Decreased in ESS
Song et al., 2019 (36)	RCT	rTMS	Right posterior parietal cortex	1 HZ, 34 min, 14 day, at daytime	ESS	Declined in ESS
Jiao et al., 2020 (37)	RCT	ta-VNS	ABVN	Repeated protocol of 20 Hz for 10 s and 4 Hz for 5 s, for 30 min per session, two sessions per day, 5 days per week for 4 weeks, applied in the morning and about half an hour bedtime	ESS	Decreased in ESS
OSA						
(38–49)	retrospective and prospective study	HNS	Hypoglossal nerve	Delivering stimulation pulses between the end of expiration and the beginning of the next expiratory phase of each respiratory cycle	ESS	ESS was reduced
Steffen et al., 2023 (50)	Case	HNS, PNS	Hypoglossal nerve, phrenic nerve	–	ESS	ESS daytime sleepiness improved
Narcolepsy or idiopathic hypersomnia (IH)						
Hohenester et al., 2023 (51)	RCT	tDCS, tRNS	Anode on the FP1 and FP2, cathode on the P3 and P4	tDCS: 2 mA, 2 × 13 min, 3 day, at daytime tRNS: same setup with 100−640 Hz was used	ESS	No significant reduce in ESS
Galbiati et al., 2016 (52)	RCT	tDCS	Anode on the F3, cathode on the contralateral orbit	2 mA, 20 min, three per week, 4 weeks, at daytime	ANT, ESS	Reduced in EDS
Lai et al., 2017 (53)	Case	rTMS	F3	10 HZ, 5 per week, 5 weeks, at daytime	ESS	Reduced in ESS
Winter et al., 2024 (54)	prospective study	VNS	Vagus nerve	0.25 mA, 30 HZ signal Off-time of 5 min and signal On-time of 30 s. the output current during the day time was increased by 0.25 mA every 2 weeks until of 2.0 mA	ESS	Significant improvement on ESS after three and six months

### 3.3 Effects of neuromodulation on EDS in patients with medical or neurologic disease

Chronic sleepiness is common in patients with organic damage. A case report provided initial evidence that bifrontal anodal tDCS (FP1, FP2) alleviates daytime drowsiness in a patient post-reanimation (55). A 52-year-old man who had been in good health until a severe allergic reaction 10 years earlier developed EDS following reanimation. Despite extensive treatment, no substantial improvement occurred. The patient’s medications were not changed. The patient underwent two blocks comprised of three anodal tDCS stimulation sessions, followed by 1 month. Subjective vigilance (VAS) and self-reported daytime sleep duration were evaluated pre- and post-intervention. The findings indicated a substantial increase in VAS and a reduction in reported daytime sleep duration over the study period (55). Another phase of the research showed immediate vigilance enhancement (PVT-measured) post-anodal stimulation.

Insomnia, hypersomnia, and parasomnia are common phenomena in patients with Parkinson’s disease (PD), which contributes to EDS (56). Only two studies determined the influence of tES on EDS in patients with PD. A randomized, double-blind, parallel-group trial was conducted involving 23 PD patients, who were divided into groups receiving tDCS combined with occupational therapy or sham tDCS with occupational therapy. It was shown that tDCS had a significant effect on fatigue. Although there was a reduction in the average ESS score from pre-treatment to immediately post-treatment in the experimental group, the differences were not significant (57). The results were consistent with previous studies on the effects of low-amperage alternating current stimulation on the psychological symptoms of early PD (58). The probable reason for the low initial ESS scores could be the baseline score. Therefore, future research with a larger sample size focusing on patients with more pronounced daytime sleepiness is recommended.

Three studies evaluated the impact of rTMS on PD patients with EDS. Low frequency rTMS over the right DLPFC has been shown to effectively improve EDS in patients with PD (59). In this parallel, sham-controlled study, 25 PD patients with potential EDS, as indicated by ESS scores, were randomly assigned to an active or sham group. The active group received 1 Hz rTMS over 10 consecutive days with ESS assessments at the start, post-treatment, and 1 month later. The active group showed a significant reduction in ESS scores at both follow-ups compared to baseline (59). In addition, the degree of ESS score change was shown to correlate positively with the duration of the disease during the follow-up period. In contrast, another study demonstrated the beneficial effects of bilateral motor cortex (M1) high-frequency rTMS on depression and quality of life in PD patients but no improvement in sleepiness (60). When the same stimulation parameters of rTMS over the left DLPFC were applied, no alleviation in EDS occurred (61).

DBS is an effective method for the treatment of middle and advanced PD. Subthalamic nucleus (STN)-DBS is the most frequently used. Several studies have reported an improvement in EDS after STN-DBS in PD patients (62–65), while other studies detected no significant changes (66–70). The reasons for the discrepant results might be associated with the limited number of participants and the diversity in subjective symptom evaluation. Additionally, one study showed that pedunculopontine nucleus (PPN)-DBS improves EDS (71). Indeed, among PD patients who received simultaneous STN and PPN-DBS, PPN-DBS led to a notable enhancement in nocturnal sleep and a substantial reduction in daytime sleepiness (71).

Patients with epilepsy often complain of drowsiness. A prospective study evaluated the effects of VNS on daytime sleepiness in patients with drug-resistant epilepsy (72). Moreover, daytime sleepiness symptoms decreased slightly after VNS treatment, although the change was not statistically significant (72). However, three earlier studies concluded that VNS significantly reduces daytime sleepiness in patients with epilepsy (73–75). Moreover, two of the studies objectively determined that VNS significantly improved the mean sleep latency at low stimulus intensities using a multiple latency sleep test (MLST), indicating reduced daytime sleepiness (73, 75).

EDS and fatigue are also frequently reported in patients with multiple sclerosis (MS) (76). A pilot study with a small sample size explored the effect of bifrontal tDCS on sleep in MS patients (77). Three patients received active tDCS and four patients received sham tDCS. The authors concluded that the ESS was significantly decreased after tDCS treatment (77). As a result, it was suggested that bifrontal tDCS resulted in significant improvement in daytime sleepiness, which was consistent with the finding that tDCS improves fatigue in MS patients (76).

A recent study evaluated the effectiveness of taVNS in patients with mild cognitive impairment (MCI) (78). In the taVNS group a pair of auricular acupoints were stimulated, including the heart (concha [CO15]) and kidney (CO10), in the distribution of the vagus nerve. In the sham taVNS group, another pair of auricular acupoints was stimulated, including the elbow (scaphoid fossa [SF3]) and shoulder (SF4, 5), in the distribution of vagus nerve. In both groups, the ESS had a significant reduction post-intervention, although there were significant differences when the difference before and after intervention were compared in the taVNS and sham taVNS groups (78).

In conclusion, among the studies related to EDS in patients with PD, no significant improvements were noted for tDCS, tACS, and high frequency rTMS, while low frequency rTMS at F4 showed positive results and the improvement in EDS lasted at least 1 month (**Table 3**) (59). These discrepancies could be attributed to variations in stimulation intensity and the specific stimulation sites that were used. Therefore, further research is warranted to evaluate these factors and the implications for treatment efficacy. DBS had been shown to alleviate the motor symptoms in patients with PD, improve the overall sleep quality, and reduce EDS. Of note, most DBS sleep studies have outcomes with <1 year of follow-up evaluations. However, some studies indicated that DBS does not significantly affect daytime sleepiness. For EDS in patients with epilepsy, low VNS stimulus intensities may lead to improvement in EDS. For EDS in patients with organic damage or MS, tES might be a treatment option to consider.

**Table 3:** Effects of neuromodulation on EDS in patients with medical or neurologic disease.

Study	Study design	Method	Target area	Protocol	Assessment	Results
Patients with organic damage						
Frase. et al., 2015 (55)	Case report	tDCS	Anode on the FP1 and FP2, cathode on the P3 and P4	2 mA, 2 × 13 min, 3 days, at daytime	PVT, VAS, self-report	Decreased sleepiness
Patients with PD						
Forogh. et al., 2016 (57)	RCT	tDCS	Anode electrode was placed over F3, cathode electrode was placed over F4	0.06 mA, 20 min, 8 sessions, 2 weeks, at daytime	ESS	No significant differences in ESS
Holly et al., 2011 (58)	RCT	tACS	Forehead	15 mA, 77.5 HZ, 45 min, 5 per week, 2 weeks, at daytime	ESS	No significant differences in ESS
Zhang et al., 2022 (59)	RCT	rTMS	F4	1 HZ, 10 days, at daytime	ESS	Decreased in ESS
Makkos et al., 2016 (60)	RCT	rTMS	Bilateral M1	5 HZ, 10 days, at daytime	ESS	No significant differences in ESS
Pal et al., 2010 (61)	RCT	rTMS	F3	5 HZ, 10 days, at daytime	ESS	No significant differences in ESS
Bjerknes et al., 2022 (62), Bargiotas et al., 2017 (63), Chahine et al., 2011 (64), Cicolin et al., 2004 (65)	retrospective and prospective study	DBS	STN	–	ESS, PDSS	Improvement in EDS
Jung et al., 2020 (66), Choi et al., 2019 (67) Kharkar et al., 2018 (68) Chou et al., 2012 (70)	prospective study	DBS	STN	–	ESS, PDSS	No change in EDS
Peppe et al., 2012 (71)	prospective study	DBS	PPN, STN	–	ESS, PDSS	A significant amelioration of daytime sleepiness
Patients with epilepsy						
Kim et al., 2022 (72)	prospective study	VNS	Vagus nerve	0.25 mA, 30 Hz, on/off cycles of 30 s on and 5 min off. The output current was gradually increased at each visit (i.e., every 2−4 weeks) over several weeks from 0.25 to ≥ 0.5 mA	ESS	No significant change in ESS
Galli et al., 2003 (73), Malow et al., 2001 (75)	prospective study	VNS	Vagus nerve	0.75−2.75 mA, 30 Hz, signal off-time of 5 min and signal on-time of 30 s	MSLT	Low stimulus intensities (≤1.5 mA) significantly improved the mean sleep latency
Rizzo et al., 2003 (74)	prospective study	VNS	Vagus nerve	0.75−3.25 mA, 30 Hz signal off-time of 3−5 min and signal on-time of 30−60 s	Sleep-wake diary	A decrease in both nocturnal sleep and daytime naps and an increased daytime alertness
Patients with MS						
Chalah et al., 2022 (77)	RCT	tDCS	Anode electrode was placed over F3, cathode electrode was placed over F4	2 mA, 20 min, five daily sessions, at daytime	ESS	Decreased in ESS
Patients with MCI						
Wang et al., 2022 (78)	RCT	taVNS	Distribution of vagus nerve	20 Hz for 10 s and 4 Hz for 5 s, 30 min per session, two sessions every day, once in the morning, the other in the evening, 5 days per week for 2 weeks	ESS	Significantly decreased the ESS score

### 3.4 Effects of neuromodulation on EDS in patients with substance abuse disorders

In a randomized double-blind, phase 1 clinical trial with a sham-controlled design, 17 inpatients with cocaine use disorder (CUD) were assigned to receive real- versus sham-tDCS (2 mA for 20 min) of the right anodal/left cathodal DLPFC for 15 sessions over 5 weeks (three times/week). CUD patients who underwent real-tDCS experienced reduced sleepiness compared to patient in the sham-tDCS group (79). However, the real-tDCS effect was not sustained at the 1-month follow-up evaluation.

### 3.5 Effects of neuromodulation on EDS in patients with mood disorders

#### 3.5.1 Bipolar disorder *patients*

Hypersomnia has a complex association with mood disorders, especially atypical depression (80). Anode tDCS significantly reduced the Pittsburgh Sleep Questionnaire Index (PSQI) total score and all PSQI subdomains, including sleep duration, in euthymic bipolar patients. Hypersomnia is defined as an increased propensity for falling asleep or an increased sleep duration. Decreased sleep duration was indirectly shown to improve daytime sleepiness in patients with bipolar disorder (81). The same results were reported in a subsequent study that investigated the effects of tDCS on sleep quality and symptoms of depression in patients with insomnia (82).

#### 3.5.2 Depressed *patients*

An open-label study examined the effects of high frequency rTMS on sleep disturbances in adolescents with major depressive disorder (83). Seventeen patients received 10 Hz rTMS on the left DLPFC in 30 sessions. The main finding was a significant main effect of time on the Quick Inventory of Depressive Symptomatology-Adolescent (17 item) Self-report (QIDS-A17-SR) hypersomnia score, with significant improvement from baseline to 10 treatments and from baseline to 6-month follow-up evaluation (83). Based on exploratory sensitivity analyses, response/non-response to rTMS for overall depressive symptoms had no significant effect on sleep outcomes. Therefore, rTMS may have intrinsic effects on hypersomnia apart from the antidepressant effects in depressed adolescents. In another study of depressed patients after a traumatic brain injury, 1 Hz rTMS at the right DLPFC had minor improvement in sleepiness (84).

To summarize, anodal tDCS on the left DLPFC might be effective for EDS in patients with bipolar disorder or depression (**Table 4**). High frequency of left DLPFC or low frequency of right DLPFC rTMS improves EDS in patients with depression. Although ECT is very effective in treating mood disorders, especially mood disorders accompanied by suicidal thoughts, in the current research on the treatment of mood disorders with ECT there was no evaluation of the impact on EDS. However, some studies have shown that ECT improves sleep problems caused by mood disorders (85).

**Table 4:** Effects of neuromodulation on EDS in patients with mood disorders.

Study	Study design	Method	Target area	Protocol	Assessment	Results
Bipolar disorder patients						
Minichino et al., 2014 (81)	RCT	tDCS	Anode over the F3, cathode on the right cerebellar cortex	2 mA, 20 min, 3 weeks, at daytime	PSQI	Reduced in sleep duration
Depression patients						
Zhou et al., 2020 (82)	RCT	tDCS	Anode over the F3, cathode on the F4	2 mA, 30 min, 20 sessions,4 weeks, at daytime	PSQI	Reduced in sleep duration
Sonmez et al., 2019 (83)	RCT	rTMS	F3	10 HZ, 5 per week, 6 weeks, at daytime	QIDS-A17-SR	Significant main effect on QIDS-A17-SR hypersomnia scores
Rao et al., 2019 (84)	RCT	rTMS	F4	1 HZ, 5 per week, 4 weeks, at daytime	PSG, ESS	Minor improvement in sleepiness

### 3.6 Comparative efficacy of neuromodulation techniques

The findings from the studies reviewed in sections 3.1 to 3.5 are synthesized in **Table 5**, which provides a comparative overview of the evidence for EDS improvement across different neuromodulation techniques and patient populations. This summary highlights the techniques and conditions where the most consistent benefits have been reported, as well as areas with mixed or limited evidence. The table serves to illustrate the current distribution of research efforts and findings, setting the stage for a critical discussion of the strengths and limitations of the existing literature in the following section.

**Table 5:** Comparative assessment of neuromodulation techniques for EDS across patient populations.

Patient population	tDCS	tACS	rTMS	taVNS	HNS	DBS	VNS
Healthy (Sleep Deprivation)	+++ (25–27)	–	–	–	–	–	–
Insomnia	+/- (32, 33)	+ (34)	+ (36)	+ (37)	–	–	–
Obstructive Sleep Apnea (OSA)	–	–	–	–	+++ (38–49)	–	–
Narcolepsy/Idiopathic Hypersomnia	+/- (51, 52)	–	++ (53)	–	–	–	++ (54)
Parkinson’s Disease (PD)	– (57)	– (58)	+ (59) *¹	–	–	+/- (62–71)	–
Epilepsy	–	–	–	–	–	–	+ (72–75)
Organic Brain Injury/Multiple Sclerosis	+ (55, 77)	–	–	–	–	–	–
Substance Use Disorder	+ (79) *²	–	–	–	–	–	–
Mood Disorders	+ (81, 82)	–	+ (83, 84)	–	–	–	–

*¹ Effect shown specifically with 1 Hz stimulation targeting the right dorsolateral prefrontal cortex.

*² Short-term improvement; effect not sustained at 1-month follow-up.

Evidence Level Legend:

+++: Strong Evidence. Consistent, positive results from multiple studies.

++: Moderate Evidence. Positive evidence from a few controlled studies.

+: Preliminary Evidence. Suggestive findings from small-scale or open-label studies.

+/-: Inconclusive Evidence. Mixed or conflicting results reported.

–: No Evidence. No studies or no significant evidence found for EDS in this population.

References are provided in parentheses.

## 4 Discussion

EDS is becoming increasingly prevalent, with a wide range of underlying factors contributing to its occurrence. Despite the growing recognition of EDS, effective pharmacologic treatments are limited. However, neuromodulation therapies have shown promising results in addressing this challenge. This review summarizes existing research, predominantly focusing on the application of NIBS techniques in EDS, particularly highlighting the use of tDCS and rTMS. Anodal tDCS (F3/F4/FP1/FP2, 1–2 mA) or high frequency tACS has been shown to reduce sleepiness and increase vigilance in cases of EDS caused by a variety of disorders. High frequency rTMS (10 Hz) over the left DLPFC and low frequency rTMS (1 Hz) at the right DLPFC or posterior parietal cortex have demonstrated beneficial outcomes in EDS treatment. Invasive neuromodulation techniques, DBS, has positive effects on sleep regulation in PD patients and may contribute to improving EDS. Similarly, VNS has demonstrated potential benefits for patients with epilepsy, particularly patients experiencing EDS or co-morbid narcolepsy.

### 4.1 NIBS

Studies using tES and rTMS both suggested the possibility of altering brain function noninvasively in connection with sleep and alertness through targeted adjustments in the frequency of neuronal discharges (10). Specifically, tES with anodal tDCS or high frequency tACS are techniques capable of reducing sleepiness and enhancing vigilance by diminishing low frequency neuronal activity and simultaneously boosting higher frequencies (14, 86). It is important to note that the majority of the referenced studies utilized anodal tDCS to achieve the treatment goals. This approach is favored because anodal stimulation boosts cortical activity, which in turn leads to cortical desynchronization and promotes alertness (87). This finding is particularly relevant to EDS, in which increasing cortical excitability during the day might help reduce sleepiness. In this process, increased neurochemicals, including N-acetylaspartate, glutamate, and glutamine, appear to have an important role (88). Furthermore, selecting the frontal cortex as the target for modulating sleepiness seems appropriate, aligning with the critical function of the frontal cortex in initiating sleep and promoting slow-wave sleep (89). For example, anodal tDCS over F3 and/or F4 provoked active condition, which was characterized by the localized increase of higher frequency bands (α, β1, and β2) and spread reduction in slow EEG frequencies (δ and θ) (25, 90). Gamma-band EEG power during rest anticipates polarity-specific alterations in cortical arousal, serving as a possible neural mechanism for the impact of tDCS on sleep (91). In like manner, applying bifrontal tDCS 1 h before bedtime enhances wakeful EEG gamma power and reduces total nocturnal sleep duration (28). In addition, 30 Hz tACS blocks drowsiness, while boosting delta power and marginally elevating gamma power (92). Moreover, tES offers advantages in performance for complex and dynamic activities, such as navigation and driving. Research has utilized EEG-gyroscope-tDCS brain machine interfaces for preemptive management of driver sleepiness (93). Neuroimaging data indicated superior target detection with tDCS over placebo, with cerebral blood flow velocity decreasing less and oxygenation increasing more as task duration increased (94). Such results are encouraging for the potential of tDCS to counteract performance decline in attention-demanding work environments. It is also critical to consider the technical specifics of bilateral anodal tDCS setups. The configuration for delivering current to dual anodes (e.g., F3/F4 or FP1/FP2) can vary significantly. A common method involves using a Y-cable from a single-channel stimulator (28), creating a parallel circuit that splits the total current (e.g., 2 mA total, providing ~1 mA per anode). In contrast, a series connection or, more appropriately, using a stimulator with independent dual-anode channels can deliver the intended current (e.g., 2 mA) to each anode independently. This distinction is crucial as it directly impacts the current density and the resultant cortical excitability. Nevertheless, in many publications, these details are not explicitly reported (77). One might think that a parallel connection, providing a current of only 1 mA, could be responsible for the lack of treatment effectiveness in some tDCS investigations. Future research should clearly specify the stimulation setup and may benefit from using tDCS devices with independent dual-output channels for bilateral protocols to ensure precise and adequate current delivery to each target.

Neuronal activity is stimulated across synapses directly by rTMS. Typically, rTMS at frequencies >5 Hz enhances cortical excitability, while frequencies <1 Hz reduce cortical excitability (95). Research has shown that high frequency (10 Hz) rTMS applied to the left DLPFC decreased alpha power during REM sleep, suggesting an increase in cortical activity (96). Decreased slow wave sleep (SWA) has been shown in some depressed patients; hypersomnia potentially compensates for this finding by extending sleep duration (97). Hypersomnia is alleviated by increasing SWA following rTMS treatment, thereby reducing the need for compensatory sleep (98). Additionally, low frequency rTMS (1 Hz) applied to the right DLPFC may improve EDS symptoms in patients with PD, although the precise mechanism is still under investigation (59). The right DLPFC is a melatonin-sensitive area, the dysfunction of which is linked to PD patients with EDS. The level of right DLPFC activity is closely associated with the melatonin level (99). Therefore, low frequency rTMS might alleviate PD patients with EDS by reducing abnormal activation of the right DLPFC related to melatonin circadian dysfunction. Primary insomnia (PI) is a condition characterized by abnormal brain network connectivity. Targeting the right posterior parietal cortex with 1 Hz rTMS has shown promise in treating PI by normalizing the temporal dynamics of EEG networks (36). Overall, rTMS shows promise for improving various clinical symptoms, including EDS. Further investigation into different rTMS protocols to enhance EDS treatment is warranted.

Currently, only five studies have assessed the aftereffects of NIBS on EDS. One study focused on tDCS and shown that the benefits of tDCS were not sustained following 1-month post-treatment (79). The four studies examining the aftereffects of rTMS demonstrated that these effects could potentially last up to 1 month (36, 59), 6 months (83), or even 1 year (53). However, specific outcomes may vary due to multiple factors and further research is ongoing to determine the duration of effects in specific individuals and treatment contexts.

Compared to rTMS and tES, ECT is a longer and more effective technique to treat schizophrenia and mood disorders (85). However, EDS symptoms associated with these disorders after ECT have not been reported. Although narcolepsy is not an indication for ECT, major depression secondary to narcolepsy responds well to ECT (100). Considering that the presence of narcolepsy facilitates the side effects of psychotropic drugs, such as stimulants exacerbating hallucinations in patients with narcolepsy and comorbid mental illness, ECT is the treatment of choice.

Another non-invasive technique that modulates arousal pathways is Electrical Vestibular Stimulation (VeNS). VeNS delivers low-intensity electrical currents via electrodes placed on the mastoid processes to stimulate the vestibular system. Although direct studies on VeNS for EDS are currently lacking, its potential relevance stems from its influence on brainstem structures integral to the ascending reticular activating system (ARAS), such as the locus coeruleus and raphe nuclei, which are crucial for sleep-wake regulation (101). Importantly, recent randomized, sham-controlled trials have demonstrated the efficacy of VeNS in improving insomnia severity—a condition closely linked to daytime dysfunction (101, 102). Given that improving nocturnal sleep quality is a fundamental strategy for mitigating EDS, these findings position VeNS as a promising neuromodulation approach worthy of future investigation specifically for disorders of excessive daytime sleepiness.

Furthermore, an emerging technique of significant promise is temporal interference stimulation (TI), which enables non-invasive and focal stimulation of deep brain or peripheral nerve targets by utilizing the interference pattern of two high-frequency electric fields. Proof-of-concept studies have demonstrated its capability to modulate hippocampal activity in humans and, when applied bilaterally to the hypoglossal nerves, to reduce the apnea-hypopnea index in patients with obstructive sleep apnea (103, 104). Although not yet applied directly to EDS, TI’s unique ability to overcome the depth-focality trade-off of conventional non-invasive techniques positions it as a compelling tool for future research, particularly for targeting subcortical sleep-wake centers or peripheral nerves implicated in EDS pathophysiology.

Beyond primary sleep disorders, NIBS techniques have shown therapeutic promise across a range of neurological and psychiatric conditions—such as depression, Parkinson’s disease, Alzheimer’s disease, and multiple sclerosis—many of which are frequently comorbid with sleep disturbances (105). In these disorders, NIBS may improve sleep quality and daytime alertness by modulating cortical excitability, neurotransmitter balance, and large-scale brain networks involved in arousal regulation. These findings highlight the potential transdiagnostic utility of NIBS for managing EDS in the context of complex, multimorbid patient populations, warranting further investigation into individualized stimulation approaches.

### 4.2 Invasive neuromodulation

Invasive neuromodulation bypasses the limitations of NIBS by providing direct neural activation and reaching deeper brain regions through implanted devices. Sleep-wake disturbances (SWDs) are prevalent nonmotor issues in PD patients (106). Several theories have been advanced to explain the improvement in SWD following DBS. DBS may exert an indirect effect by alleviating nocturnal motor complications and reducing the reliance on dopaminergic medications (22). Alternatively, DBS can improve sleep-wake regulation through a circuit-mediated direct effect (107). Under physiologic conditions, the ventral tegmental area (VTA) of the midbrain, which is rich in dopaminergic neurons, receives regulatory input from orexin neurons in the hypothalamus. This interaction forms a descending regulatory circuit that encompasses the cortex, thalamus, and brainstem nuclei, all of which have critical roles in sleep and wakefulness (106). Given the neural projections from the STN to sleep-regulating areas, such as the cortex, thalamus, and pontine nuclei, it is plausible that the STN is intricately involved in the modulation of sleep-wake functions, thereby influencing sleep-wake disorders. Research has demonstrated that STN-DBS not only alleviates motor symptoms but also enhances overall sleep quality and reduces EDS (22). However, some studies have indicated that STN-DBS does not significantly alter levels of daytime sleepiness, suggesting that the mechanisms contributing to EDS in this patient population are likely multifactorial. Factors, such as disease progression, medication interactions, and individual variability in neural circuitry, may all have a role in the persistence or variability of sleep outcomes post-DBS. Moreover, most studies investigating the DBS impact on sleep have outcomes with follow-up periods <1 year. This limitation raises several critical questions regarding the longevity of the benefits provided by STN-DBS: How long do these improvements last? Will the beneficial effects persist over time or will the beneficial effects diminish? Additionally, what specific factors are correlated with postoperative sleep improvement? Addressing these questions through long-term studies will be essential for optimizing DBS protocols and enhancing the quality of life for patients with PD experiencing SWD.

VNS is an established treatment for diseases, such as epilepsy and depression (23). The observation of increased alertness and reduced sleepiness during VNS therapy in epilepsy patients has prompted investigations into the effectiveness in narcolepsy, in which the initial evaluation has shown significant improvement in daily sleepiness (54). This makes VNS a potential additional treatment for narcolepsy patients who do not respond well to medication. The mechanism by which VNS affects EDS is complex. In narcolepsy, the absence of orexin-producing cells in the lateral hypothalamus leads to less activity in areas that maintain the awake state, like the locus coeruleus and the basal forebrain (108). VNS stimulates orexin neurons, promoting wakefulness and thereby reducing daytime sleepiness (109). In mouse model study, VNS activates parts of the brain that use noradrenaline and acetylcholine, causing widespread arousal and increased alertness (110). Noninvasive transcutaneous VNS represents a significant advance, allowing for stimulation of the vagus nerve without the need for surgical intervention. This noninvasive approach is particularly appealing for patients with psychiatric disorders because the noninvasive approach broadens the applicability of VNS therapy and enhance patient adherence.

In conclusion, both DBS and VNS highlight the intricate relationship between neuromodulation and sleep. These therapies not only target alleviation of motor symptoms in PD or seizure reduction in epilepsy but also offer additional advantages by addressing the sleep disturbances commonly associated with these conditions. However, further studies are required to thoroughly understand the complex interplay of mechanisms involved and to assess the long-term sustainability of these benefits.

### 4.3 Commercially available devices and clinical translation

The translation of neuromodulation research into clinical practice is evidenced by the availability of several commercially approved devices. For instance, the Inspire^®^ system is an FDA-approved hypoglossal nerve stimulator for the treatment of moderate to severe obstructive sleep apnea (46), which indirectly addresses EDS by improving underlying sleep-disordered breathing. Non-invasive transcutaneous auricular VNS (taVNS) devices are also being developed and marketed for conditions like epilepsy and depression, with growing interest in their application for sleep disorders (111). Additionally, wearable VeNS devices have emerged as consumer wellness products aimed at improving sleep and relaxation, although robust clinical evidence for their efficacy in EDS is still evolving (101, 102). Acknowledging these developments is crucial, as it bridges the gap between experimental findings and tangible therapeutic options available to clinicians and patients. The clinical status and evidence level for EDS of various techniques are summarized in **Table 6**. This translation to commercially available options highlights the growing practical relevance of neuromodulation for sleep-wake disorders.

**Table 6:** Overview of neuromodulation techniques for EDS.

Technique	Typical target	Evidence level for EDS	Representative clinical/regulatory status
tDCS	Prefrontal cortex	Moderate (various etiologies)	Investigational device; research use
rTMS	Left DLPFC (10 Hz) or right DLPFC (1 Hz)	Moderate (PD, depression, insomnia)	FDA-cleared for depression, OCD; EDS use remains investigational
taVNS	Auricular concha	Emerging (insomnia)	CE-marked devices available; FDA investigational for various conditions
VeNS	Vestibular system (mastoid)	Emerging (insomnia)	FDA-approved for chronic insomnia (Modius Sleep); consumer wellness devices available
HNS	Hypoglossal nerve	High (OSA-associated EDS)	FDA-approved (Inspire^®^) for moderate-to-severe OSA
DBS	STN/PPN (PD)	Moderate (PD-associated EDS)	FDA-approved for Parkinson’s disease, essential tremor
VNS	Vagus nerve	Emerging (epilepsy, narcolepsy)	FDA-approved for epilepsy, treatment-resistant depression

### 4.4 Ethical considerations, long-term safety, and challenges in clinical translation

The clinical translation of neuromodulation for EDS is challenged by significant ethical, safety, and practical hurdles. Ethically, the substantial risks of invasive techniques demand careful risk-benefit justification for a non-life-threatening condition, raising concerns about patient selection, cognitive enhancement, and obligations in sham-controlled trials. Furthermore, long-term safety profiles for both non-invasive and invasive approaches remain inadequately characterized, necessitating dedicated monitoring. Practically, widespread adoption is hindered by a lack of protocol standardization, regulatory barriers due to insufficient evidence, and challenges related to cost, training, and reimbursement, which ultimately limit patient access.

### 4.5 Limitations and future perspectives

While the existing body of research provides promising evidence for the role of neuromodulation in alleviating EDS, it is imperative to acknowledge the methodological limitations that temper the strength of these conclusions. A substantial proportion of the cited studies are characterized by small sample sizes, lack of sham-controlled or blinded designs, heterogeneous stimulation parameters, and reliance on subjective outcome measures (e.g., ESS scores) rather than objective polysomnographic or neurophysiological data. For instance, many tDCS and rTMS trials included in this review enrolled fewer than 30 participants, limiting statistical power and generalizability. Furthermore, the predominance of open-label studies and case reports, particularly in investigations of invasive techniques, introduces potential bias and precludes causal inference.

In direct response to these challenges, future research must transition from proof-of-concept demonstrations to the establishment of clinically robust, evidence-based protocols. This evolution should be guided by the following specific and actionable priorities:

1. Enhancing Methodological Rigor and Objective Validation. The foremost priority is the execution of adequately powered, randomized, double-blind, sham-controlled trials. To fully realize the potential of neuromodulation, future studies must prioritize larger sample sizes and refinement of stimulation parameters (e.g., frequency, duration, intensity). Crucially, these trials must prioritize the use of objective biomarkers (e.g., PSG, actigraphy, EEG-based vigilance measures) as primary endpoints and adhere to standardized protocols (e.g., dose defined by individualized electric field modeling) to facilitate replication and meta-analysis.
2. Elucidating Mechanisms and Optimizing Protocols. Research must move beyond empirical observation to mechanistic understanding. This includes using neuroimaging (fMRI, PET) to validate target engagement and delineate the neural circuits modulated by treatment. Concurrently, comparative effectiveness studies are needed to identify optimal stimulation sites (e.g., comparing DLPFC with other cortical targets) and parameters, and to explore synergies with adjunctive pharmacological therapies.
3. Paving the Way for Personalization and Expanding Therapeutic Boundaries. A critical frontier is the development of predictive models for treatment selection. Integrating explainable machine learning (XML) with multimodal data (clinical, genetic, neurophysiological) will be essential to identify predictors of response, ultimately enabling a shift from a trial-and-error approach to tailored, precision neuromodulation (112). Furthermore, the therapeutic landscape should be broadened by investigating invasive options (e.g., DBS, VNS, HNS) for treatment-resistant cases, particularly for conditions like narcolepsy or idiopathic hypersomnia where evidence is currently limited, while carefully weighing the associated ethical and surgical risks. The exploration of novel non-invasive techniques, such as temporal interference (TI) stimulation for deep brain targets, also represents a promising avenue.

By systematically addressing these limitations and pursuing these targeted research directions, the field can solidify the role of neuromodulation as a validated and personalized therapeutic strategy for EDS, moving it from a promising intervention to an integral component of the clinical toolkit.

## 5 Conclusions

In conclusion, the burgeoning field of noninvasive neuromodulation for EDS presents a promising horizon of opportunities to enhance current therapeutic modalities. However, the small sample sizes and the preliminary nature of existing studies underscore the need for larger, well-controlled trials to confirm these initial observations. Furthermore, research is essential to refine treatment protocols and validate outcomes through objective measures. Investigating invasive options for persistent cases may also broaden the therapeutic landscape for EDS. This review contributes to the growing body of knowledge on neuromodulation for EDS, providing a foundation for future research and the development of personalized, effective interventions.
